# Draft Genome Sequence of *Gardnerella vaginalis* Strain ATCC 49145 Associated with Bacterial Vaginosis

**DOI:** 10.1128/genomeA.00286-17

**Published:** 2017-05-04

**Authors:** Destaalem T. Kidane, Brock A. Arivett, Jacob Crigler, Eric J. Vick, Anthony L. Farone, Mary B. Farone

**Affiliations:** a Biology Department, Middle Tennessee State University, Murfreesboro, Tennessee, USA; b College of Medicine, University of Tennessee Health Science Center, Memphis, Tennessee, USA

## Abstract

*Gardnerella vaginalis* is a Gram-variable bacterium associated with bacterial vaginosis, a common vaginal inflammation in women of reproductive age. This study reports the whole-genome sequencing for the clinical isolate strain ATCC 49145. The draft genome is composed of 21 contigs containing 1,325 protein-coding sequences, 45 tRNAs and a single tmRNA (SsrA).

## GENOME ANNOUNCEMENT

*Gardnerella vaginalis*, the only member of the genus *Gardnerella*, is a nonmotile facultative anaerobic Gram-variable bacterium associated with bacterial vaginosis (BV), pelvic inflammatory disease, and preterm birth. Originally isolated in 1953 by Leopold (1) and then characterized in 1955 by Gardner and Dukes (2), it is associated with the human female reproductive tract and makes a human-specific cytolysin, suggesting coevolution. It is well known that *G. vaginalis* forms DNA-dependent biofilms, which enhance the resistance of the microbe to oral metronidazole (3). The pathogenicity of *G. vaginalis* in humans is often subject to debate, but the bacterium has been reported as causing isolated cases of osteomyelitis, urethritis, and bacteremia (4). Notably, colonization with *G. vaginalis* and recurrent bacterial vaginosis is associated with preterm birth and pelvic inflammatory disease (5). Recent reports have suggested that *G. vaginalis* may have a more pathogenic role than opportunistic flora and may directly contribute to a proinflammatory environment within the female genital tract by increasing production of cytokines and invading the epithelial surface (6). The genome of the clinical isolate *G. vaginalis* ATCC 49145 was sequenced using next-generation sequencing methods in order to compare this strain with other catalogued strains and further elucidate how *G. vaginalis* may act as both a benign commensal and a pathogenic organism.

For whole-genome sequencing, total DNA from *G. vaginalis* strain ATCC 49145 was isolated from colonies grown overnight on brain heart infusion agar (Becton, Dickinson, Franklin Lakes, NJ, USA) at 37°C and 5% CO_2_ using the DNeasy blood and tissue kit (Qiagen, Valencia, CA, USA). DNA was cleaned with a Zymo genomic DNA clean and concentrator kit (Zymo, Irvine, CA, USA). Absorption was measured at ratios of 260/280 and 260/230 to estimate DNA quality and quantity using a NanoDrop 2000 (Thermo Scientific, Wilmington, DE, USA). Whole-genome sequencing was performed using the 250-bp paired-end MiSeq platform (Illumina, San Diego, CA, USA) maintained at the Department of Biology, Middle Tennessee State University (Murfreesboro, TN, USA) after the construction of a paired-end sequencing library using the Illumina Nextera XT DNA sample preparation kit (Illumina, San Diego, CA, USA). Assessment of the quality of the read data was conducted using the FASTQC software program (http://www.bioinformatics.babraham.ac.uk/projects/fastqc/). The reads were assembled *de novo* using Genomics Workbench version 8.0 with the Bacterial Genome Finishing module (CLC Bio, Boston, MA, USA). The final assembly consisted of 1,706,848 bp in 21 contigs, with an average G+C content of 41.2% and an *N*_50_ contig size of 159,097 bp. Functional genome annotation was performed using Prokka version 1.10 (7) on a Quad-Core i7 workstation with 32-gigabyte DDR3 running Ubuntu version 14.04 LTS. The annotation of *G. vaginalis* strain ATCC 49145 resulted in the identification of 1,371 open reading frames, consisting of 1,325 protein-coding sequences, 45 tRNAs, and a single tmRNA (SsrA) sequence.

### Accession number(s).

This whole-genome shotgun project has been deposited at DDBJ/ENA/GenBank under the accession number MJBN00000000. The version described in this paper is the first version, MJBN01000000.
